# GREB1 is an estrogen receptor-regulated tumour promoter that is frequently expressed in ovarian cancer

**DOI:** 10.1038/s41388-018-0377-y

**Published:** 2018-07-04

**Authors:** Kendra Hodgkinson, Laura A. Forrest, Nhung Vuong, Kenneth Garson, Bojana Djordjevic, Barbara C. Vanderhyden

**Affiliations:** 1 Department of Cellular and Molecular Medicine, University of Ottawa, Ottawa, ON Canada; 2 Centre for Cancer Therapeutics, Ottawa Hospital Research Institute, Ottawa, ON Canada; 3 Department of Pathology and Laboratory Medicine, The Ottawa Hospital, University of Ottawa, Ottawa, ON Canada

## Abstract

Estrogenic hormone replacement therapy increases the risk of developing ovarian cancer, and estrogen promotes tumour initiation and growth in mouse models of this disease. GREB1 (Growth regulation by estrogen in breast cancer 1) is an ESR1 (estrogen receptor 1)-upregulated protein which may mediate estrogen action. GREB1 knockdown prevents hormone-driven proliferation of several breast and prostate cancer cell lines and prolongs survival of mice engrafted with ovarian cancer cells, but its mechanism of action remains unclear. In this study, we explored GREB1 function in ovarian cancer. GREB1 overexpression in ovarian cancer cell lines increased cell proliferation and migration and promoted a mesenchymal morphology associated with increased *Col1a2*, which encodes a collagen I subunit. GREB1 knockdown inhibited proliferation and promoted an epithelial morphology associated with decreased *Col1a2*. In human tissues, GREB1 was expressed in all ESR1-expressing tissues throughout the normal female reproductive tract, in addition to several tissues that did not show ESR1 expression. In a TMA of ovarian cancer cases, GREB1 was expressed in 75–85% of serous, endometrioid, mucinous, and clear cell carcinomas. Serous, endometrioid, and mucinous ovarian cancers were almost always positive for either ESR1 or GREB1, suggesting a possible reliance on signalling through ESR1 and/or GREB1. Targeting GREB1 may inhibit tumour-promoting pathways both downstream and independent of ESR1 and is therefore a possible treatment strategy worthy of further investigation.

## Introduction

Ovarian cancer has a high mortality rate, often attributed to the fact that 75% of patients are diagnosed at advanced stages [1]. There are few effective treatments or screening methods for ovarian cancer [2]. The tissues of origin and early stages of disease progression are still controversial. However, histological and molecular comparisons and the identification of precursor lesions in high-risk women and transgenic mouse models have indicated that ovarian cancer can derive from at least three different epithelial tissues: the ovarian surface epithelium (OSE), the fallopian tube epithelium, and mislocalized endometrial tissue (endometriosis) [3].

Studying the risk factors for ovarian cancer can provide insight into early disease progression. Epidemiological and experimental evidence show that estrogen (17β-estradiol; E2) promotes ovarian tumorigenesis and cancer progression predominantly through the estrogen receptor ESR1 [4, 5]. A recent meta-analysis revealed that risk was notably increased for two common subtypes of epithelial ovarian cancer (EOC): serous and endometrioid [6]. Furthermore, anti-estrogen maintenance therapy prolonged survival of low-grade serous ovarian cancer patients (from 26.4 to 64.9 months) [7].

ESR1 signalling in ovarian cancer involves many interconnected pathways including IL6/STAT3, PI3K/AKT, and MAPK1, making it difficult to elucidate the mechanisms directly responsible for E2-stimulated tumour growth [4, 5]. The PI3K/AKT and MAPK1 pathways contribute to cancer cell proliferation, survival, metastasis, and invasion, and AKT is often overexpressed in EOC [8]. E2 promotes migration and invasion in several ovarian cancer cell lines [9, 10] and can also induce epithelial-to-mesenchymal transition (EMT), as shown by changes in morphology, *SNAI1* induction, and decreased *CDH1* (E-cadherin) [11, 12]. Furthermore, E2 actions in vivo are not always seen in vitro [13, 14], suggesting that E2 can promote tumour growth through systemic actions [15], tumour microenvironment, and/or differential effects on tumour cells growing in vitro vs. in vivo. Therefore, animal models are required to elucidate the complex actions of E2 in ovarian cancer. E2 promotes ovarian cancer growth in many xenograft and transgenic models [13, 16–20], but the mechanisms underlying this accelerated growth remain unclear.

The mechanism of E2 action can be elucidated by examining genes altered by E2 treatment in mouse models. Microarray analysis identified *Greb1* (Growth regulation by estrogen in breast cancer 1) as a highly E2-upregulated gene in tumours from an E2-responsive mouse model of ovarian cancer [14]. *GREB1* expression correlates with ESR1 positivity in breast cancer cell lines and primary breast tumours [21–24], and *GREB1* is induced by ESR1 binding to estrogen response elements (EREs) upstream of the *GREB1* promoter [25, 26]. *GREB1* is also induced by E2 through MYC-mediated downregulation of miR-26 [27]. GREB1 is required for hormone-stimulated growth in breast and prostate cancer cells [22, 28] and is a cofactor for ESR1 transcriptional activity [24], but its function otherwise remains unknown.

We showed previously that stable GREB1 knockdown in mouse ovarian cancer cells reduces proliferation and prolongs survival of engrafted mice [14]. Given that *GREB1* induction is dependent on E2 signalling, we used the CAG-TAg murine model of ovarian cancer [13] to determine the impact of *Esr1* deletion on survival of mice with and without exogenous E2 treatment. We also further investigated the function of GREB1 by examining GREB1 constitutive expression or knockdown and cell morphology, EMT, and migration in vitro, and on tumorigenicity in allograft models of ovarian cancer in vivo. GREB1 protein expression was reported previously only in breast [23] and uterine [29] tissues; however, *GREB1* mRNA was highly expressed in ovarian cancers [14]. We have therefore examined *GREB1* mRNA expression in public databases and GREB1 protein expression in tissue microarrays (TMAs) of normal tissues and EOCs of all major histological subtypes. Any possible correlation between ESR1 and GREB1 expression was also examined to determine whether GREB1 correlates with ESR1 in ovarian cancer, as reported in breast cancer [23].

## Results

We showed previously that exogenous E2 accelerates tumour progression using MASE cells grafted into SCID mice, reducing median survival time by 55% [14]. Gene expression analysis of the tumours showed that ESR1 likely mediates this effect, as *Esr1* was highly expressed in both control and E2-stimulated tumours relative to normal ovary, whereas *Esr2* was expressed at much lower levels in MASE-derived tumours (Figure S1).

**Fig. 1:**
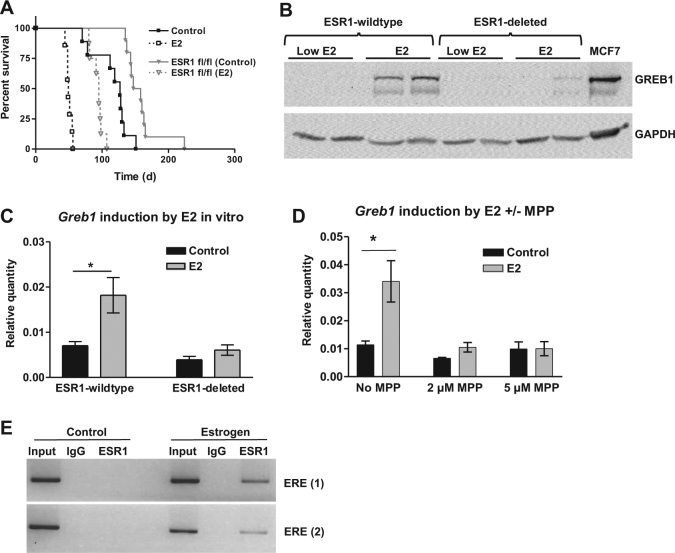
Role of ESR1 in tumour progression and *Greb1* induction. **a** *Esr1* deletion in the tumour-initiating cells prolongs survival and abrogates E2 response of CAG-TAg mice (*N* = 8–10; log rank test). **b** GREB1 is detectable by western blot in tumours of mice implanted with 0.25 mg E2 pellets but not in mice implanted with low-dose E2 (0.05 mg) or (not shown) placebo pellets. *Esr1* deletion inhibits GREB1 expression. **c** *Greb1* is induced by E2 treatment in a primary ascites cell line derived from *Esr1*-wildtype but not in a matched cell line derived from *Esr1*-deleted mice (*n* = 3; *t*-test). **d** *Greb1* induction by E2 in another ascites cell line is prevented by ESR1 inhibition (MPP; *n* = 3; *t*-test). **e** Chromatin immunoprecipitation shows ligand-dependent ESR1 binding to two EREs associated with the *Greb1* promoter. *p < 0.05, **p < 0.01, ***p < 0.0001

To determine the importance of ESR1 for E2-accelerated tumorigenesis, CAG-TAg transgenic mice were crossed with ESR1-floxed mice [30] to generate CAG-TAg mice homozygous for the floxed *Esr1* allele. *Esr1* was then deleted concurrently with TAg activation in the OSE by intrabursal AdCre. E2 decreased survival time by 61% in mice with wild-type *Esr1*, and by 38% in mice with *Esr1* deleted in the OSE (Fig. 1a), showing that ESR1 partially mediates E2 tumour promotion in this mouse model. Furthermore, we found that endogenous E2 also promotes tumour growth through ESR1 in this model; ESR1 inactivation prolonged median survival by 20% even in mice not treated with hormones (*p* = 0.0003). Tumour burden, ascites volume, and ascites incidence were not changed by E2 or *Esr1* deletion (Figure S2).

Microarray analysis of E2-stimulated tumours identified *Greb1* as a highly upregulated gene [14], and we therefore investigated its role in ESR1-mediated tumour growth. GREB1 E2-induction in tumours was inhibited by *Esr1* deletion (Fig. 1b) and MASE cell lines derived from the ascites of mice with *Esr1* deletion did not show *Greb1* induction with E2 treatment in vitro (Fig. 1c). Inhibiting ESR1 activity with a specific antagonist (methyl-piperidino-pyrazole; MPP) prevented *Greb1* E2-induction at the mRNA level (Fig. 1d) and protein level (Figure S3). Using chromatin immunoprecipitation (ChIP) to determine if *Greb1* transcription is induced by ESR1 binding to the *Greb1* promoter region, both EREs examined showed clear ligand-dependent ESR1 binding in MASE cells (Fig. 1e).

**Fig. 2:**
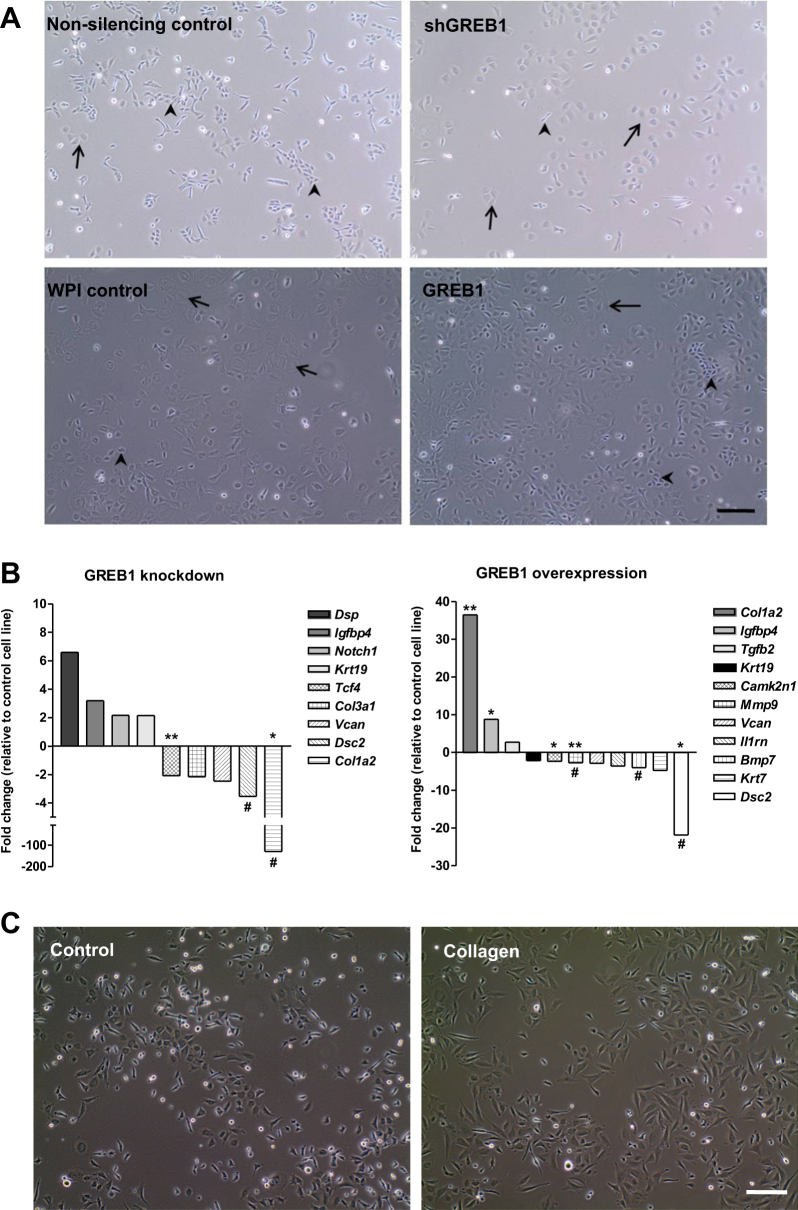
Effect of GREB1 knockdown and overexpression on MASE morphology and EMT. **a** Shift in proportions of large, cobblestone “epithelial-like” cells (arrows) vs. small, fibroblastic “mesenchymal-like” cells (arrowheads) after GREB1 knockdown (top) and overexpression (bottom). Scale bar: 100 µm (same magnification for all panels). **b** Gene expression changes (two-fold or higher) in a RT^2^ Profiler PCR array of EMT markers (*n* = 3). **c** MASE cell morphology was more mesenchymal-looking when grown on tissue culture plates coated with collagen I. *p < 0.05, **p < 0.01 (*t*-test). #: Cycle threshold above 30 after GREB1 knockdown or overexpression

To investigate GREB1 function, stable knockdown and overexpression of GREB1 was achieved using lentiviral constructs in MASE cells. Following selection, a stable difference in morphology was seen with both GREB1 knockdown and overexpression. Although cell lines remained a mix of morphologies, clear changes were observed in the proportion of each phenotype. GREB1 knockdown showed an increased proportion of large, round cells in tight clusters and a decreased proportion of smaller, more extended cells (Fig. 2a). GREB1 overexpression caused a morphology shift in the opposite direction. Supporting these observations, average cell volumes were decreased 32% by GREB1 overexpression (*p* = 0.0016) and showed a trend for 148% increase with GREB1 knockdown (*p* = 0.0756; Figure S4).

To determine whether these effects on morphology were caused by an EMT, a panel of 80 EMT-related genes were examined with a QPCR-based RT^2^ profiler array. Most genes, including the canonical EMT markers *Snai1* and *Cdh1*, were not altered by GREB1 modulation. However, six genes showed opposite trends with GREB1 knockdown vs. overexpression (*Bmp7*, *Col1a2*, *Gng11*, *Krt19*, *Notch1*, and *Tcf4*; Fig. 2b). Only *Col1a2*, a mesenchymal marker which encodes a subunit of collagen I, significantly changed in both directions, with GREB1 knockdown downregulating *Col1a2* more than 99% (*p* = 0.011717), and GREB1 overexpression increasing *Col1a2* 37-fold (*p* = 0.001515; Fig. 2b). *Krt19*, an epithelial marker encoding keratin 19 (a type I keratin), was decreased with GREB1 overexpression and increased with GREB1 knockdown. A significant decrease in expression of *Bmp7* (Bone morphogenetic protein-7) in GREB1-overexpressing cells agrees with its reported role in maintaining the epithelial phenotype [31]. Together, these trends suggest that GREB1 overexpression is associated with a more epithelial phenotype, while GREB1 knockdown causes a mesenchymal phenotype. Interestingly, plating MASE cells on collagen I-coated plates promoted a morphology similar to that seen with GREB1 overexpression, suggesting that the morphology changes observed with GREB1 modulation are mediated at least in part by changes in collagen I production (Fig. 2c). This was confirmed by overexpression of *Col1a2* in MASE cells, which resulted in a notable shift to a more mesenchymal phenotype, although this did not alter cell proliferation or migration (Figure S5).

**Fig. 3:**
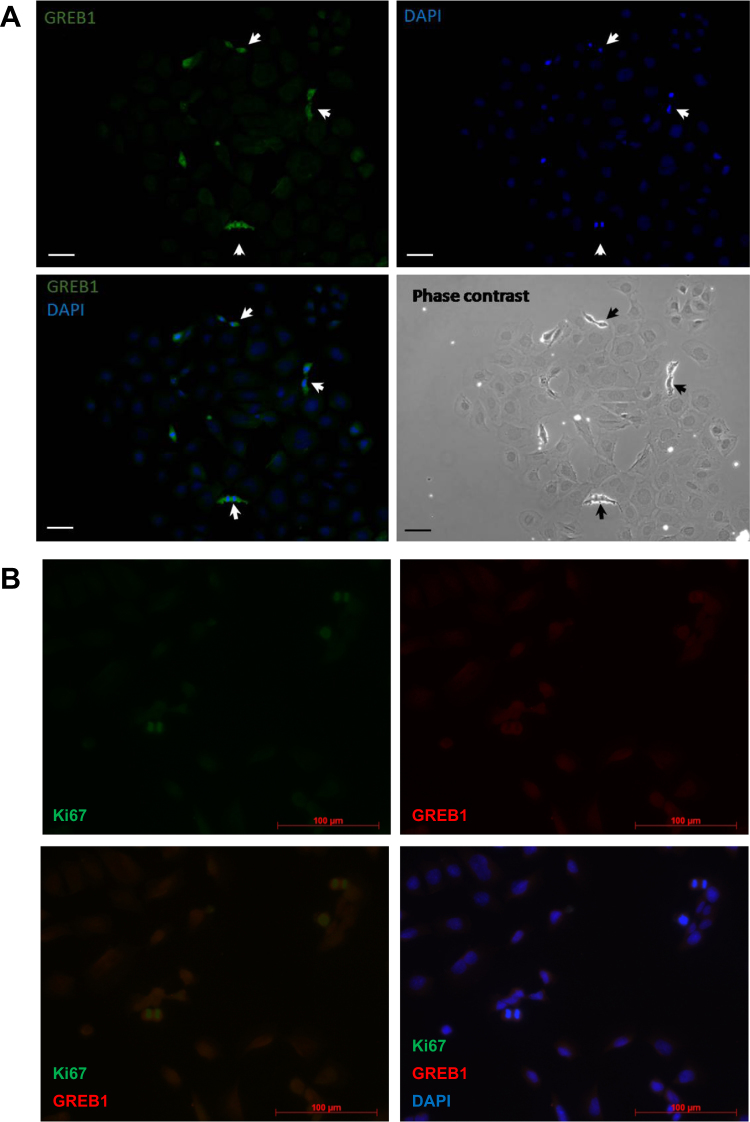
GREB1 association with mitosis in nontransduced MASE cells. MASE cells were treated with 10 nM E2 for 48 h (**a**) or cultured in α-MEM containing 10% FBS (**b**), then fixed and examined by immunofluorescence. **a** The subpopulation of MASE cells with highest GREB1 expression (arrows) showed a mitotic morphology and their DAPI staining indicates highly condensed DNA. Scale bar: 50 µm. **b** The subset of MASE cells showing high expression of the proliferation marker KI67 (green) also show high GREB1 expression (red) and condensed DAPI staining (blue)

These morphology changes may also be related to proliferation, as GREB1 was associated with a distinctly mitotic morphology in MASE cells (Fig. 3a). Cells expressing higher levels of GREB1 by immunofluorescence were a small subpopulation that appeared fibroblastic or mesenchymal, were small and rounded, had DAPI staining that indicates condensed chromatin, and were frequently seen in pairs, suggesting recently completed mitosis. These cells were also typically the most strongly KI67-positive cells in the field of view (Fig. 3b).

**Fig. 4:**
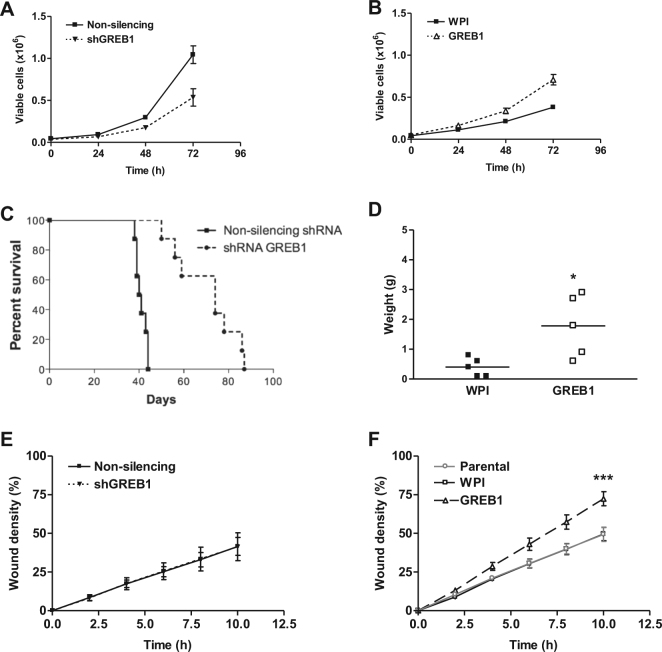
Effect of GREB1 modulation on MASE cell proliferation and migration. **a** Proliferation was decreased by GREB1 knockdown and **b** increased by GREB1 overexpression (*n* = 3). **c** In mice engrafted with MASE cells, GREB1 knockdown prolonged survival and **d** GREB1 overexpression increased tumour growth (*n* = 5–8). **e**, **f** Migration of MASE cells was assessed by scratch wound assays; GREB1 knockdown (**e**) did not alter wound closure, but GREB1 overexpression (**f**) increased the rate of wound closure (*n* = 3; linear regression). **a** and **c** reproduced with permission.^6^ *p < 0.05, ***p < 0.0001 (*t*-test comparing doubling times)

To clarify the link between high GREB1 expression and mitosis, the effect of GREB1 modulation on proliferation was examined. In low-serum conditions, GREB1 knockdown showed slower proliferation (*p* = 0.0460; Fig. 4a) and GREB1 overexpression showed more rapid proliferation (*p* = 0.0435; Fig. 4b). In 10% serum, GREB1 knockdown did not alter proliferation (not shown). Substrate-independent growth was assessed by growth of single cells suspended in soft agar; surprisingly, colony formation was not altered by GREB1 knockdown or overexpression (not shown).

When these cells were engrafted into SCID mice, GREB1 knockdown almost doubled median survival time (Fig. 4c). Tumour weight at endpoint was not altered by GREB1 knockdown, implying that the knockdown slowed tumour growth. GREB1 overexpression increased average tumour weight at endpoint 4.5-fold (Fig. 4d) but surprisingly did not alter survival, perhaps because the average volume of ascites in these mice was lower than in control mice (0.62 ± 0.88 vs. 5.30 ± 2.41 ml).

To determine whether GREB1 alters migration, a scratch wound assay was performed. GREB1 knockdown in MASE cells in serum-containing media did not alter migration (Fig. 4e), but GREB1 overexpression increased average migration rate by 45% in low-serum media (*p* < 0.0001; Fig. 4f). Similarly, GREB1 overexpression increased migration rate by 57% in E2-free media (*p* < 0.0001; Figure S6), and by 27% with a 10 nM E2 treatment (*p* = 0.03111).

**Fig. 5:**
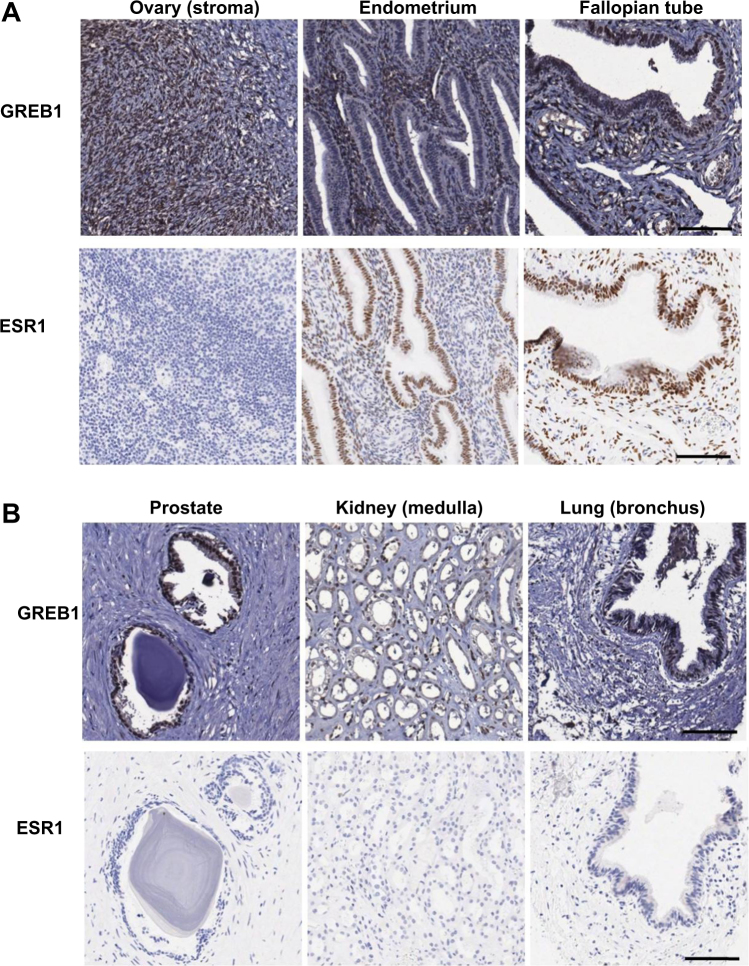
GREB1 and ESR1 expression in normal tissues on a tissue microarray. **a** GREB1 was highly expressed throughout the female reproductive tract, including the ovary, endometrium, and fallopian tube. ESR1 was also expressed in most female reproductive tissues including the endometrium and fallopian tube. **b** GREB1 was expressed in several tissues outside the female reproductive tract, including prostate, kidney, and lung; ESR1 was not expressed in these tissues. Scale bar: 100 µm

GREB1 protein expression is reported in normal breast and uterine tissues [12, 18, 21] but not in other tissues. RNA-sequencing data accessed through the EMBL-EBI Expression Atlas indicated that *GREB1* mRNA levels are highest in the ovary, with moderate expression in the prostate and female reproductive tissues and much lower but still detectable expression in all other tissues examined except tonsil and whole blood. To assess GREB1 expression in normal tissues, we used immunohistochemistry (IHC) to examine GREB1 and colocalization with ESR1 in a TMA panel of 50 normal tissues. GREB1 was highly expressed throughout the female reproductive tract, including the ovarian stroma, fallopian tube (stroma and epithelium), uterus (smooth muscle and endometrium), and cervix (ectocervix and endocervix); ESR1 colocalized with GREB1 in most of these tissues (Fig. 5a). GREB1 was also expressed in several tissues outside of the reproductive tract, including hormone-responsive tissues (breast, prostate) and others (bronchus epithelium, thyroid, salivary gland, and kidney medulla); these tissues were almost all ESR1− (Fig. 5b). The only tissue with no detectable *GREB1* mRNA, tonsil, also showed no detectable GREB1 protein. In this TMA, ESR1+ tissues were all GREB1+, and many ESR1− tissues were also GREB1+.

**Fig. 6:**
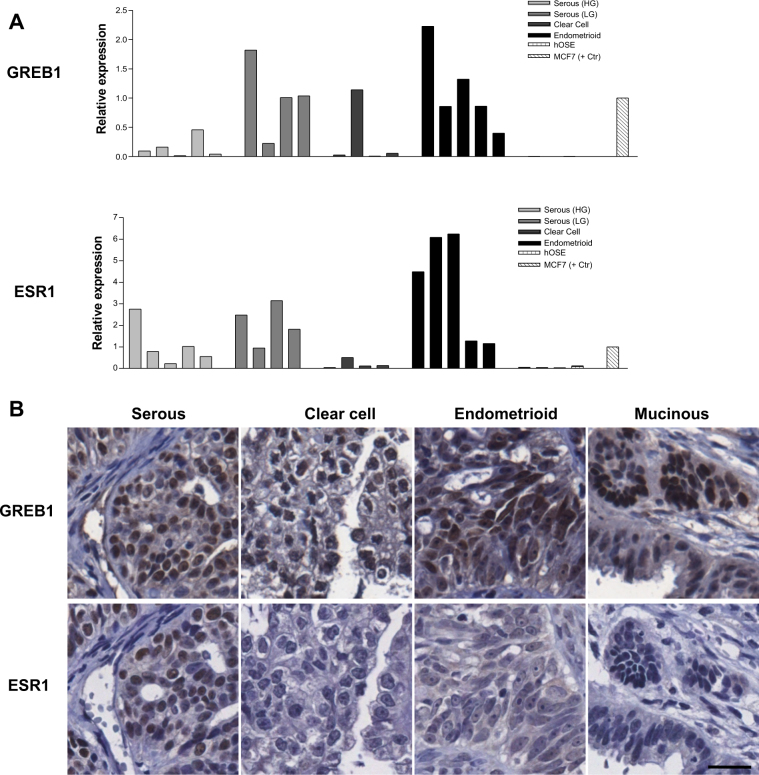
GREB1 and ESR1 expression in epithelial ovarian cancers. **a** *GREB1* and *ESR1* mRNA were examined by QPCR in snap-frozen ovarian tumours, with gene expression normalized to PPIA (*N* = 4–5 per subtype). Very low expression was seen in four early-passage cultures of human ovarian surface epithelial cells. **b** In a tissue microarray of four major EOC histological subtypes (*N* = 20 each), GREB1 was expressed with similar proportions across all subtypes and did not correlate with ESR1 expression (see Fig. 7). Scale bar: 25 µm. **a** reproduced with permission^6^

**Fig. 7:**
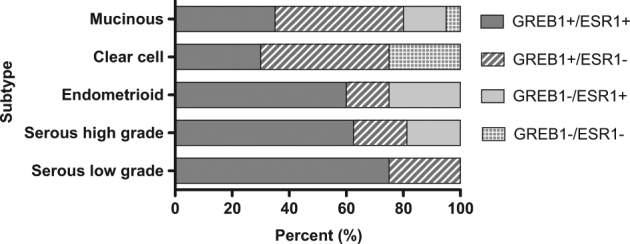
GREB1 and ESR1 expression in a tissue microarray of tumours from the five major histological subtypes of ovarian cancer. The number of tumours that fall into each category (GREB1+/ESR1+, GREB1+/ESR1−, GREB1−/ESR1+, GREB1−/ESR1−) is expressed as a percentage. GREB1 expression is represented by shades of grey (dark grey GREB1+, light grey GREB1−) and ESR1 expression by patterns (ESR1+ slashes, ESR1− dots)

To investigate the possible role of *GREB1* in human ovarian cancers, we explored its expression using a public database (TCGA; via cBioPortal). *GREB1* was highest in hormone-responsive cancers (ovarian, uterine, breast, prostate) and unexpectedly, melanoma. The lowest expression was seen in leukaemia and digestive tract cancers. Similar expression patterns were seen for *ESR1*, with highest expression in breast, ovarian, and uterine cancers, and lowest expression in colorectal cancers. Notably, cBioPortal and similar databases focus on the most common subtype of EOC (high-grade serous), and very little information is available for other subtypes. We therefore investigated GREB1 expression by QPCR and IHC in tumours of all major histological EOC subtypes. GREB1 mRNA and protein were frequently expressed in all subtypes investigated; ESR1 and GREB1 correlated at the mRNA level (Fig. 6a) but not at the protein level (Fig. 6b). In fact, 81% of ESR1− and 78% of ESR1+ tumours were GREB1+ by IHC, with no major differences across subtypes. GREB1 showed a trend for more frequent expression in low-grade tumours and in younger women, but sample sizes were too small to draw clear conclusions. Most serous and endometrioid tumours were ESR1+, whereas the percentage was reduced for mucinous (50% ESR1+) and clear cell tumours (30%) (Fig. 7).

## Discussion

E2 signalling complexity and heterogeneity between model systems have made it difficult to determine the molecular mechanisms responsible for its tumour-promoting actions. We previously described many gene expression changes that may mediate the tumour-promoting effects of E2 in our models of EOC [14]. One of these E2-regulated genes, *GREB1*, is particularly promising based on its role in E2-stimulated breast cancer cell proliferation [22]. Herein we have further explored the effects of GREB1 knockdown and overexpression in ovarian cancer cells. We found that in mouse ovarian cancer cells, *Greb1* is induced by E2 through ESR1 binding to EREs, as shown previously in breast cancer cells [25, 26]. ESR1 inhibition or deletion prevented GREB1 upregulation both in vitro and in vivo. *Esr1* deletion in tumours prolonged survival and decreased, but did not eliminate, E2 responsiveness. The ESR1-driven subset of E2 actions may be partially or entirely mediated by GREB1, which drives proliferation and tumour growth in mouse models of ovarian cancer.

Modulation of GREB1 also altered morphology. Surprisingly, canonical EMT markers were not altered by GREB1 knockdown or overexpression, but the mesenchymal marker *Col1a2* was greatly increased by GREB1 overexpression and decreased by GREB1 knockdown. *Col1a2* differential transcription is associated with an EMT in a variety of cell types, representing both fibrosis and cancer models [21, 32–35], including ovarian cancer [36]. It has been shown that transforming growth factor-β stimulates *COL1A2* transcription, and that this is associated with an EMT [32–34, 36]. The mechanisms behind this are context-dependent, with some fibrosis models citing a p300/CBP transactivation with Smads [21] and others citing Smad3-independent signalling [32]. While the mechanism remains to be elucidated, overexpression of *Col1a2* or culture on collagen I both promoted a mesenchymal morphology of MASE cells, suggesting that the morphology changes associated with GREB1 modulation are mediated by changes in *Col1a2* production. GREB1 overexpression promoted migration in all culture conditions, consistent with the change to a mesenchymal phenotype.

In vivo, GREB1 knockdown in tumours decreased survival, but surprisingly, overexpression did not alter survival. However, GREB1 overexpression accelerated tumour growth; average tumour weight at endpoint was 4.5-fold higher in mice engrafted with GREB1-overexpressing cells vs. control cells. Despite the increased tumour growth, mice had lower volumes of ascites than controls, leading to equal survival in both groups despite the differences in disease progression. It is unclear why GREB1 overexpression decreased ascites volume; additional study is required to clarify the underlying mechanisms.

In normal tissues, GREB1 expression has only been reported in breast and uterine epithelia [23, 29]. Public databases show that *GREB1* mRNA levels are highest in the reproductive tract and in cancers arising from those tissues. Our TMA panel of normal tissues showed that GREB1 expression was highest in the prostate and female reproductive tract as expected, but it was also detected in many tissues with relatively low *GREB1* mRNA levels. Tonsils had no detectable *GREB1* transcripts reported in the EMBL-EBI Expression Atlas, and showed no evidence of GREB1 protein expression in the TMA. ESR1 protein expression also resembled mRNA patterns described in public databases, with strong expression in most tissues of the female reproductive tract but rare expression elsewhere. All ESR1+ tissues expressed GREB1, but many ESR1− tissues also expressed GREB1. This suggests that GREB1 is not solely regulated by ESR1, particularly in tissues outside of the female reproductive tract.

Previous studies in endometriosis and breast cancer reported that GREB1 is mainly nuclear, which supports its role as an ESR1 cofactor [23, 24, 29]. However, we discovered that GREB1 was strongly expressed in both the nucleus and the cytoplasm of E2-treated mouse ovarian cancer cells. Interestingly, this expression was focal, and appeared to be associated with mitotic cells. It is unclear whether GREB1 is only ever expressed in a subpopulation of cells, or whether all MASE cells express GREB1 periodically (i.e. during mitosis). Regardless, this mitosis-linked expression pattern along with its ability to increase proliferation suggests that GREB1 is enhancing cell division. In contrast, more than three-quarters of human EOC tissues examined showed clear nuclear GREB1 staining. Nuclear staining was also observed throughout the reproductive tract and in other normal tissues, although cytoplasmic staining was also observed in both normal and EOC tissues. A previous report suggested that GREB1 localization may be associated with abnormal growth; GREB1 was nuclear in endometriotic lesions but cytoplasmic in normal endometrial tissue [29].

We found that *GREB1* mRNA levels correlated with *ESR1* in ovarian tumours, but GREB1 protein levels did not correlate with ESR1 in the TMA. The high-grade serous ovarian tumours from the TCGA showed no correlation between *GREB1* and *ESR1* overexpression, suggesting that the correlation we observed between *ESR1* and *GREB1* at the mRNA level may have been caused by random variation in a small sample size. Alternatively, the lack of correlation at the protein level could be caused by post-transcriptional regulation of GREB1. This difference from breast cancer [23] may reflect tissue-specific regulation of GREB1 and/or the postmenopausal status of most ovarian cancer patients. Several genes that correlate with ESR1 in breast cancer do not show a similar correlation in ovarian cancer [37], and in breast cancer, GREB1 and other E2-regulated genes correlated with serum E2 changes throughout the menstrual cycle [38]. These differences in E2-responsive genes between breast and ovarian cancers could be related to the sensitivity and resistance, respectively, of these two cancers to anti-estrogen therapies. ESR1-independent GREB1 expression could be a mechanism for stimulating growth-promoting E2 signalling pathways in the absence of E2 and/or ESR1. GREB1 loss has been linked to tamoxifen resistance in ESR1+ breast cancer and cell lines [24, 38]; however, the effects of GREB1 expression in ESR1− cell lines have not been examined.

Many ESR1+ EOC tissues did not express GREB1, whereas ESR1+ normal tissues universally expressed GREB1. Nearly all of the 60 serous, mucinous, and endometrioid tumours examined were positive for either GREB1 or ESR1 (or both); only one tumour was negative for both markers, and notably, ESR1 staining was still observed in one of three scoreable cores from this tumour. In contrast, 5 of the 20 clear cell tumours examined were negative for both GREB1 and ESR1. This suggests that serous, mucinous, and endometrioid tumours may be reliant on ESR1 and/or GREB1 function.

In this study, we have shown that E2 promotes ovarian tumour growth in mouse models and have identified GREB1 as a key mediator of this process. GREB1 promotes ovarian cancer cell proliferation and migration, possibly by upregulating the production of collagen I. In addition to expanding the knowledge of GREB1 activity by determining the effects of GREB1 interference and stable expression in several ovarian cancer model systems, we have explored its expression in ovarian cancers. GREB1 and ESR1 are co-expressed in normal female reproductive tissues, whereas GREB1 is present, with or without ESR1, in the majority of ovarian cancers of all major histological subtypes. While additional experiments are needed to elucidate the mechanism of action of GREB1, the prolonged survival of mice when GREB1 is suppressed provides initial evidence, suggesting that targeting GREB1 could have therapeutic efficacy in ovarian cancer.

## Materials and methods

### Mice

All experiments were performed according to the Canadian Council on Animal Care guidelines using protocols approved by the University of Ottawa Animal Care Committee. Mice were maintained in a 12:12 light:dark cycle with free access to food and water. ESR1^fl/fl^ TAg^+/+^ mice were generated by crossing tgCAG-LS-TAg (CAG-TAg) mice [13] with conditional *Esr1* knockout mice [30], then MAX-BAX selective backcrossing (Charles River, Montreal, Canada) was used to achieve >99% FVB/N genetic markers. Bilateral intrabursal injections of adenovirus expressing Cre recombinase (AdCre; Vector Development Laboratory, USA) were used to simultaneously activate the oncogene SV40 T antigen (TAg) and delete *Esr1* selectively in the OSE of female mice (age 8 weeks). A 60-day slow-release pellet containing 0.05 or 0.25 mg E2 or a placebo pellet were then implanted subcutaneously [13, 14]. For the allograft studies, female mice (age 6 weeks) with severe combined immunodeficiency (SCID) were obtained from Charles River. After 1 week of acclimatization, mice were anesthetised, then injected intraperitoneally with 10 million mouse ascites (MASE) cells suspended in 0.5 ml sterile phosphate-buffered saline (PBS). Surgeries were performed in alternating order between treatment groups. Survival endpoints were based on overall health assessments by veterinary technicians blinded to experimental groups. At necropsy, mice were euthanized with carbon dioxide, then tumours and ascites were removed and measured. Ascites were used to generate ascites cell lines, and tumour tissues were frozen at −80 °C or formalin-fixed and paraffin-embedded. The Animal Research: Reporting of In Vivo Experiments (ARRIVE) guidelines were followed where possible, and animals were excluded if they died from something other than tumour burden.

### Cell isolation, culture, and E2 treatments

MASE cell lines were generated from malignant ascites of E2-treated CAG-TAg mice [14]. Mouse OSE cell lines were established following isolation from normal mouse ovaries [39]. Cell lines were cultured in α-MEM (Invitrogen, USA) containing 10% FBS (Gibco, Thermo Fisher Scientific, USA). Mycoplasma testing was performed every 3 months of culture and cells tested negative. Before E2 treatments, cells were grown for 24–48 h in E2-free media: phenol-red-free DMEM/F12 (Invitrogen, USA), with 1.2 g/l sodium bicarbonate and 5–10% charcoal-stripped serum. E2 (Sigma, USA) was diluted in 100% ethanol and added at a final concentration of 10 nM. For ESR1 inhibition experiments, the ESR1-specific inhibitor methyl-piperidino-pyrazole (MPP; 1–5 µM) was added to culture media 30 min prior to E2 treatment. For collagen experiments, cells were plated on collagen I-coated plates (Corning, USA) and phase-contrast images were collected 24 h later with an EVOS microscope (Thermo Fisher Scientific, USA).

### Cell proliferation, migration, volume, and colony formation

For proliferation assays, 50,000 cells/well were plated in six-well tissue culture dishes (Corning, USA). After cell attachment (4–6 h), low-serum (1% FBS) media was used. Cells in triplicate wells were trypsinized, resuspended in α-MEM containing 10% FBS, and counted with a ViCell automated cell counter, using a trypan blue exclusion to quantify viable cells (Vi-CELL TM XR Cell Viability Analyzer; Beckman Coulter Inc., USA). Cell viability was always higher than 90%. The diameters of cells were measured with the Vi-CELL analyzer and used to calculate volume based on the approximation of a spherical cell. For migration assays, cells reached 95–100% confluency (20–24 h). Identical scratch wounds were made in every well using the IncuCyte WoundMaker (Essen BioScience, USA), then washed twice before adding treatment media. Images were taken every 2 h with the IncuCyte automated monitoring system (Essen BioScience, USA), and IncuCyte ZOOM software was used to calculate wound density (cell confluence in wound, normalized to confluence outside the wound). To assess capacity for anchorage independent growth, single-cell suspensions were prepared and colony formation in 4% serum-containing medium in soft agar was assessed and quantified after 7 days, as described previously [40].

### GREB1 knockdown and overexpression and *Col1a2* overexpression

For GREB1 knockdown, GIPZ-based lentiviral constructs encoding GREB1-targeted shRNA were obtained from Open Biosystems (now Dharmacon; USA) (see supplementary information). MASE cells were puromycin-selected 72 h after transduction of the GIPZ lentiviral vectors, then puromycin-resistant cells were GFP-sorted using fluorescence-activated cell sorting at the Ottawa Hospital Research Institute StemCore Flow Cytometry facility. For GREB1 overexpression, the coding region of the full-length isoform of human GREB1 was inserted upstream of the IRES-GFP expression cassette. Lentivirus was produced in transfected 293T cells and MASE cells transduced with the overexpression construct were GFP-sorted 72 h after transduction. For COL1A2 overexpression, the open reading frame (ORF) of murine *Col1A2* was PCR amplified from cDNA prepared from MASE cells overexpressing GREB1. The *Col1A2* ORF was inserted into lentiviral vector pWPI (pWPI was a gift from Didier Trono) (Addgene plasmid # 12254) upstream of the IRES-GFP expression cassette. MASE cells transduced with lentiviral construct were sorted for GFP expression. GREB1 knockdown and overexpression and Col1a2 overexpression were confirmed by quantitative real-time RT-PCR (QPCR) for all cell lines, as well as by western blot analysis of MASE lines (Figs. S6 and S7).

### Quantitative PCR (QPCR)

RNA was isolated from flash-frozen cell pellets and tumour tissue (GenElute: Sigma-Aldrich, USA) and cDNA was generated with 500 ng RNA using a reverse transcription kit (Bio-Rad iScript: Qiagen, USA). RNA quantity and quality were assessed with a NanoDrop ND-1000 spectrophotometer (Thermo Fisher Scientific, USA). All QPCRs were run on an ABI 7500 FAST machine (Applied Biosystems, USA) with a commercial mastermix (TAqMan: Applied Biosystems, USA; RT^2^ SYBR green: Qiagen, USA; or SsoFAST SYBR green: BioRad, USA). QPCR primers (Table S1) amplified single products of the expected size with a single-peak melt curve. Primers were further validated to ensure that primer efficiency was close to 100% and closely matched efficiency of housekeeping genes, with a dynamic range of at least three orders of magnitude. The QPCR-based RT^2^ profiler array was performed similarly, following the manufacturer’s protocol (Qiagen, USA).

### Western blots

Protein was isolated from cultured cells or homogenized tissue using M-PER cell lysis buffer (Thermo Fisher Scientific, USA). Samples were prepared for the NuPAGE electrophoresis system using manufacturer-recommended buffers with precast NuPAGE Novex 10% tris-acetate gels (Invitrogen, USA). Gels were transferred to nitrocellulose (Hybond-C Extra; Amersham Biosciences, UK), then blocked for 1 h (5% skim milk/TBST), probed with primary and horseradish peroxidase-conjugated secondary antibodies for 30 min–1 h each, and developed with the SuperSignal Western Blot Enhancer kit (Thermo Fisher Scientific, USA) and ECL Advance Western Blotting Detection Kit (Amersham Biosciences, UK). Primary antibodies detected GREB1 (1:500; Sigma HPA024616, USA), COL1A2 (1:500, Abcam 96723), and GAPDH (1:60,000; Abcam ab8245, USA).

### Chromatin immunoprecipitation

MASE cells were treated with 100 nM E2 for 45 min, then crosslinked with 1% formaldehyde, quenched with 125 mM glycine, and sonicated to produce 150–200 bp fragments. ESR1 antibody (Santa Cruz) or normal mouse Immunoglobin G control (Millipore, USA) were bound to magnetic beads (Dynabeads^®^; Thermo Fisher Scientific, USA) overnight at 4 °C. Two hundred fifty micrograms of DNA was then pre-cleared with 1 µl/ml salmon sperm DNA (Sigma, USA), 10 µl/ml ovalbumin (Sigma, USA), and 10 µl/ml magnetic beads for 1 h at 4 °C. Ten per cent of pre-cleared chromatin was saved as “input” and DNA was immunoprecipitated with antibody-bound beads overnight at 4 °C. Beads bound by immune-complexes were then collected using a Magna GrIP™ Rack (Millipore, USA) and eluted at 65 °C for 10 min in an Eppendorf Thermomixer. Reverse crosslinking and protease treatment were done on each sample. The immunoprecipitated genomic DNA fragments were isolated with phenol:chloroform:isoamyl alcohol extraction. The HotStarTaq DNA Polymerase Kit (Qiagen, USA) was used to amplify mouse *Greb1* ERE1 and ERE2 promoter regions spanning two putative EREs using primers listed in Table S1.

### Immunofluorescence

Cells were plated on sterilized glass coverslips in α-MEM containing 10% FBS. Cells treated with E2 were plated in E2-free media (see above) and treated 24 h later with 10 nM E2 for 48 h. Cells were fixed with 4% paraformaldehyde for 30 min, permeabilized with 0.2% Triton X-100 (10 min), blocked with 5% normal goat serum in PBS (1 h), and probed with primary antibody (GREB1, 1:200 Abcam or 1:100 Millipore; MKI67, aka KI67, 1:100 Millipore) for 1 h at room temperature or overnight at 4 °C. Slides were then incubated with a fluorescent secondary antibody for 1 h in the dark (AlexaFluor 488 or 594, 1:250–1:1000; Thermo Fisher Scientific, USA), then mounted with a DAPI stain (ProLong Gold Antifade Mountant; Thermo Fisher Scientific, USA) and imaged with a fluorescence microscope.

### Immunohistochemistry

TMA slides were obtained from the Cooperative Human Tissue Network (University of Virginia, USA) or the Ottawa Ovarian Cancer Tissue Bank (Ottawa, Canada) with Research Ethics Board approval and informed consent. TMA slides were deparaffinized with xylene, rehydrated with ethanol, incubated with a citrate antigen retrieval buffer (Antigen Unmasking Solution, Vector, USA) for 10 min at 100 °C, then cooled for 60–90 min. Slides were then blocked for 30–60 min (serum-free Dako protein block; Agilent, USA) and probed with primary antibody (ESR1 1:200/1 h; Abcam, USA; GREB1 1:25/2 h; Millipore, USA) diluted in Dako Antibody Diluent (Agilent, USA). Staining was visualized using Dako Envision horseradish peroxidase-conjugated reagents (30 min; Agilent, USA) followed by a 5 min incubation in a 0.2% diaminobenzidine/0.001% hydrogen peroxide solution (Sigma-Aldrich, USA). Slides were scanned with an Aperio ScanScope and images were obtained with Aperio ImageScope software (Leica Biosystems, USA). Staining was assessed by two evaluators blinded to TMA tumour type. GREB1 positivity was defined as ≥20% positive cells at intensity 1 or ≥1% positive cells at intensities 2 or 3. ESR1 positivity was defined as ≥1% positive cells regardless of intensity, according to standard practice for ESR1 scoring in breast tumours [41, 42].

### Statistical analysis

Parametric tests (Welch’s *t*-test to compare two groups, or ANOVA to compare more than two groups) were used for most experiments; nonparametric alternatives (Mann–Whitney *U*-test or Wilcoxon test) were used if distribution was non-Gaussian or included off-scale values. QPCR relative expression values were log-transformed before statistical comparison. Sample sizes were chosen to detect an effect size in line with the literature for similar models, based on standard deviations for previous studies using these methods. Graphs show means ± standard error. Results were considered statistically significant at *p* < 0.05, but raw *p-*values are also reported for transparency.

## Electronic supplementary material

Supplementary Data
