# Supplementary material for: GREB1 is an estrogen receptor-regulated tumour promoter that is frequently expressed in ovarian cancer
Source: Oncogene. 2018 Jul 4;37(44):5873–86. doi: 10.1038/s41388-018-0377-y (PMC6212416; doi:10.1038/s41388-018-0377-y)
Supplement: Supplementary file 1 — Supplementary Data [file 41388_2018_377_MOESM1_ESM.pdf]

Supplementary Data

GREB1 is an estrogen receptor-regulated tumour promoter that is frequently expressed in ovarian cancer

Kendra Hodgkinson (1,2), Laura A. Forrest (1,2), Nhung Vuong (1,2), Kenneth Garson (1,2), Bojana Djordjevic (3), Barbara C. Vanderhyden (1,2)

1. Department of Cellular and Molecular Medicine, University of Ottawa

2. Centre for Cancer Therapeutics, Ottawa Hospital Research Institute

3. Department of Pathology and Laboratory Medicine, The Ottawa Hospital, University of Ottawa

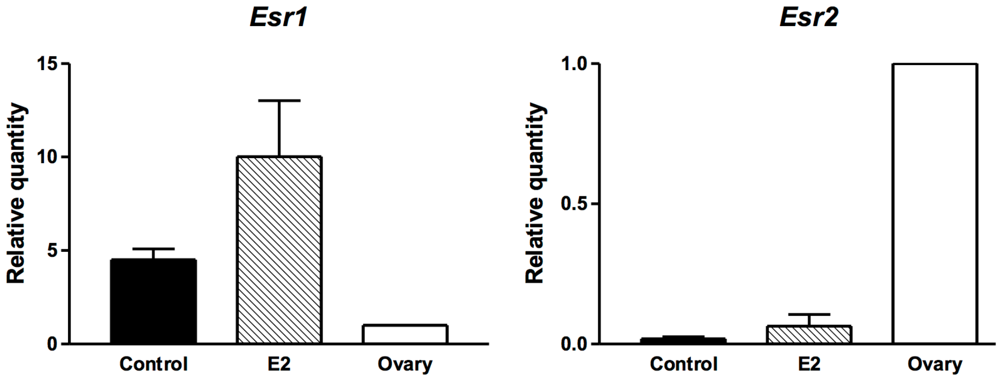

Figure S1. *Esr1* and *Esr2* expression in MASE-derived tumours. Estrogen receptors were examined by QPCR in snap-frozen MASE-derived tumours from mice described in Figure 5. *Esr1* levels were high relative to the ovary reference sample, and showed a trend for increased expression with E2 treatment (N=4-5; p=0.0495; t-test). *Esr2* levels were very low relative to the ovary sample, and showed a similar trend for increased expression with E2 (N=4-5; p=0.1768; t-test).

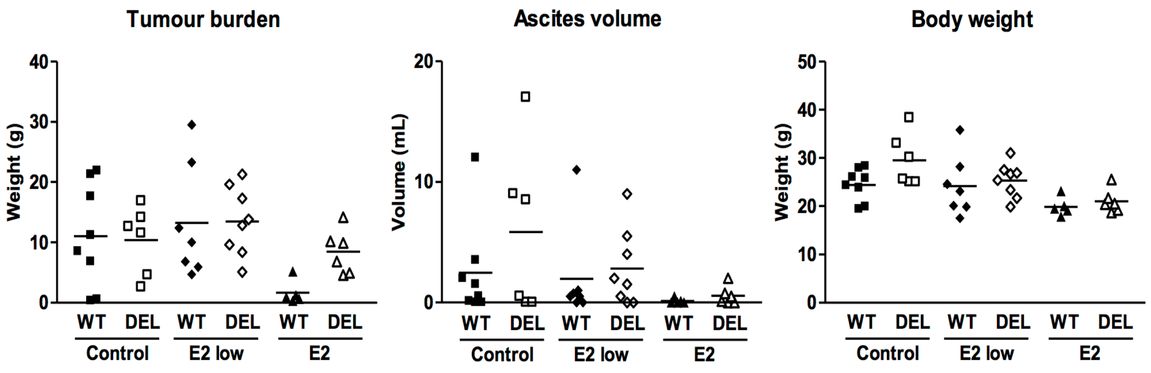

Figure S2. Tumour characteristics of CAG-TAg mice +/- ESR1 deletion and E2 treatments. CAG-TAg mice were crossed with *Esr1*-floxed mice to generate TAg-homozygous *Esr1*wt/wt and *Esr1*fl/fl siblings. At 8-10 weeks, mice were injected intrabursally with AdCre to activate TAg and delete ESR1 in the OSE, then implanted subcutaneously with an E2 (0.05 or 0.25 mg) or placebo pellet. Mice were monitored until they required a humane endpoint, then euthanized and tumour characteristics were examined. Ascites fluid (if any) was extracted through the abdominal wall with a syringe and volume measured. Tumours were then dissected out and weighed. Body weight was measured before removal of ascites and tumours; therefore, weight was corrected by subtracting tumour weight and approximate ascites weight (approximated at 1 g/mL).

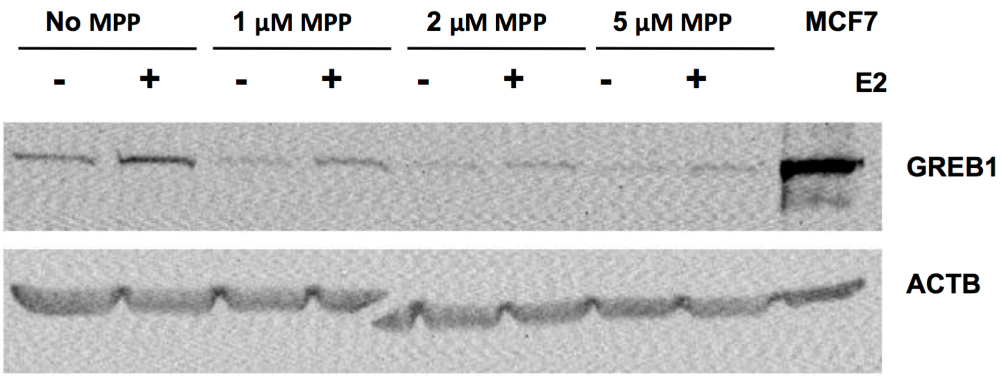

Figure S3. Confirmation at the protein level that MPP blocks GREB1 induction by E2. MASE cells were E2-deprived for 24 hours, then treated with MPP for 30 minutes before adding 10 nM E2 to each well. Protein was collected after 24 hours of E2 treatment and GREB1 levels were measured by western blot (n=2).

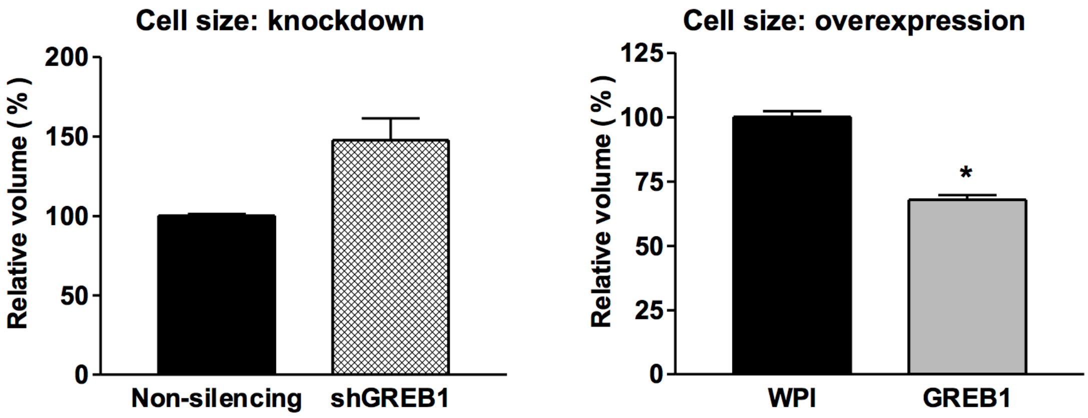

Figure S4. Cell volume changes after GREB1 knockdown and overexpression. Diameter of cells in suspension was measured with a ViCell automated counter and used to calculate approximate volume (assuming spherical cells in suspension). GREB1 knockdown showed a trend for increased cell volume (n=3; p=0.0756). GREB1 overexpression decreased cell volume (n=3; *: p<0.01; t-test).

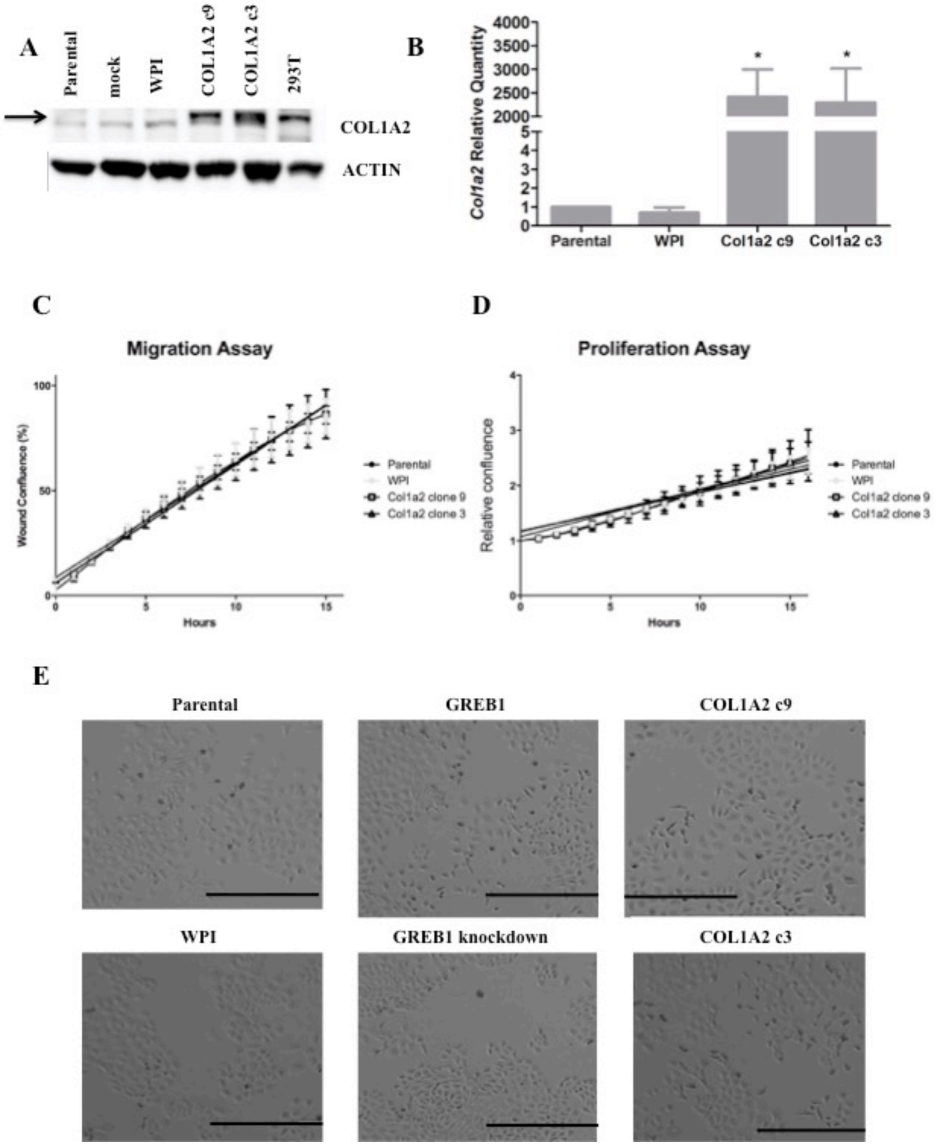

Figure S5. COL1A2 effects on MASE cell migration, proliferation, and morphology. Cells were infected with a GIPZ-based lentiviral vector containing murine COL1A2 and two clones were selected. Overexpression was confirmed via **A)** Western blot (arrow pointing to COL1A2 at 150 kDa) and **B)** qPCR. (*$p<0.05$, One way ANOVA with Dunnett's post-test, n=3). **C)** MASE parental, WPI (vector control), and the two COL1A2 overexpressing clones were plated in 96-well plates and identical scratch wounds were made in each well when cells were 100% confluent. Wells were washed twice with PBS and replenished with normal media (αMEM + 10% FBS). Wound healing was imaged and analyzed with IncuCyte ZOOM software. **D)** Cells were also plated in 96-well plates at a density of 10, 000 cells and allowed to adhere overnight. They were placed in the Incucyte the next day**.** Plate confluence was measured over a period of 48 hours and data is presented relative to time zero for each cell line**. E)** Morphology was also assessed and it was noted that the GREB1 and COL1A2 overexpressing cells have a more mesenchymal phenotype (scale bar 500 μm). WPI= vector control, 293T= positive control for COL1A2, c=clone.

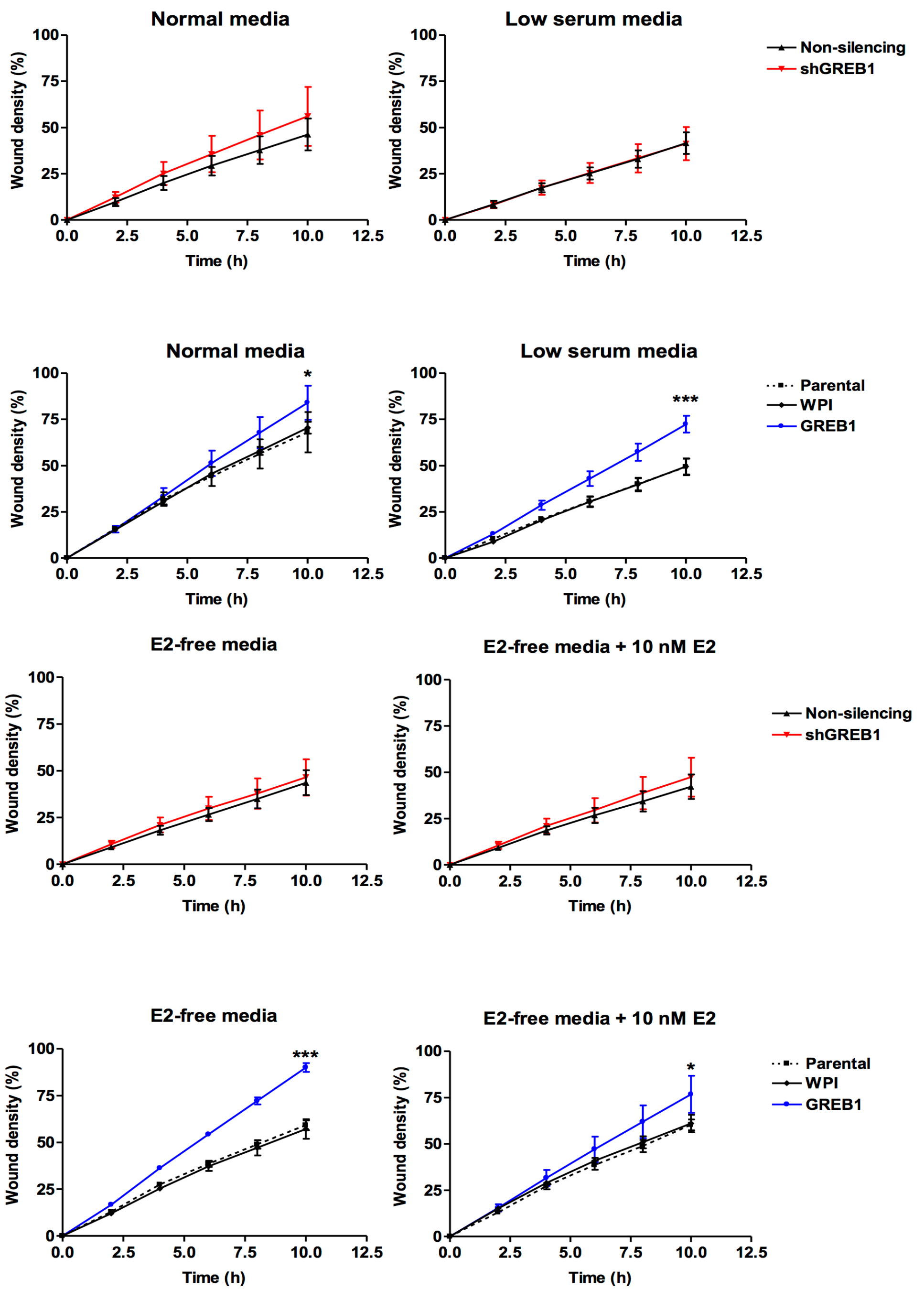

Figure S6. GREB1 effects on MASE cell migration in different media conditions. MASE cell lines with GREB1 knocked down (shGREB1) or overexpressed (GREB1) and the associated control cell lines (Non-silencing, Parental and WPI) were plated in 96-well plates and identical scratch wounds were made in each well when cells were 100% confluent. Wells were washed twice with PBS and replenished with normal media (αMEM + 10% FBS), low serum media (αMEM + 1% FBS), E2-free media (phenol-red free DMEM/F12 + 5% charcoal-stripped FBS), or E2-free media with 10 nM E2 added. Wound healing was imaged and analyzed with IncuCyte ZOOM software.

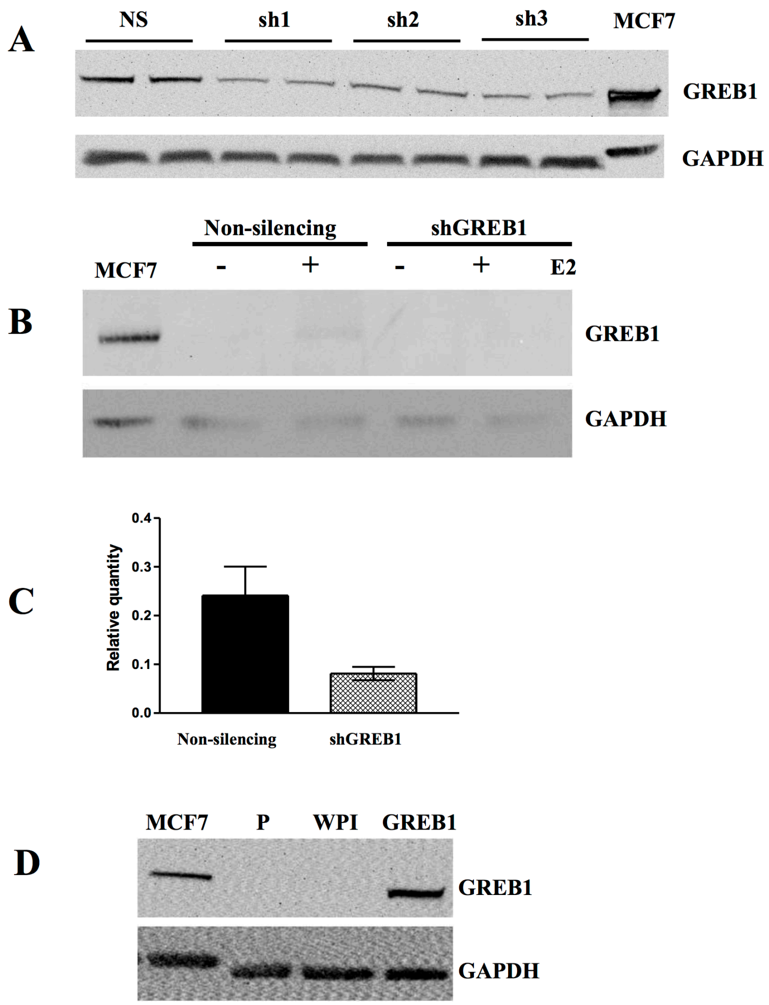

Figure S7. Confirmation of GREB1 knockdown and overexpression in MASE cells. Cells were transduced with a GIPZ-based lentiviral vector containing a short hairpin directed at GREB1 or with a WPI-based lentiviral vector containing the full-length human GREB1 sequence. Control cell lines were transduced with a non-silencing short hairpin or an empty WPI construct, respectively. Following drug selection, GREB1 baseline levels and induction by E2 were assessed by QPCR and western blot. **A)** Different short hairpin constructs were tested and all subsequent results show data from experiments using sh3 (TGCTGTTGACAGTGAGCGAATGTGACGATGTAGACTTCAAT AGTGAAGCCACAGATGTATTGAAGTCTACATCGTCACATGTGCCTACTGCCTCGGA). GREB1 levels were lower in the sh-transduced cells compared to the non-silencing control. **B)** GREB1 was induced by 500 nM E2 within 48h in non-silencing control MASE cells but not in cells transduced with the knockdown construct (shGREB1). **C)** *Greb1* mRNA levels were decreased in cells transduced with shGREB1 (n=2). **D)** GREB1 was highly expressed in MASE cells transduced with the overexpression construct (GREB1) and undetectable in the control-transduced cells (WPI) or parental MASE (P). MCF7 cells were used as a positive control.

Table S1. Primers used for QPCR and ChIP.

Target	Forward sequence	Reverse sequence
mGreb1	GCAACACGGTGCCTCCACCA	GAGGCGCCTGCTGGTACTGC
mEsr1	TCTGCAGCAGCAGCATCGCC	GGCATGAAGGCGGTGGGCAT
mEsr2	CGTTCTGGACAGGGATGAGGGGA	GGCTTGCGGTAGCCAAGGGG
mCol1a2	AAGGATACAGTGGATTGCAG	TCTACCATCTTTGCCAACGG
mPpia	AGGGTGGTGACTTTACACGC	GATGCCAGGACCTGTATGCT
hGREB1	TAGCGACCCCTGGCCAGACC	GCCGTCTGACGCCGCACATA
hESR1	CCTGATGGCCAAGGCAGGCC	CGGTGGGCGTCCAGCATCTC
hPPIA	CCTAAAGCATACGGGTCCTG	TTTCACTTTGCCAAACACCA
mERE1	ACCGCAAACTGTATCAGTGG	CCAGCTGCCACAATTAGAAAC
mERE2	ACAAACTGTCCTCACCCACAG	ACATGGGAAGCCACGATAAG

m, mouse; h, human
